# Chemical activation and magnetization of carbonaceous materials fabricated from waste plastics and their evaluation for methylene blue adsorption

**DOI:** 10.1007/s11356-024-33729-5

**Published:** 2024-07-02

**Authors:** Eslam Salama, Mahmoud Samy, Hassan Shokry Hassan, Safaa Mohamed, Kenneth Mensah, Marwa F. Elkady

**Affiliations:** 1 Environment and Natural Materials Research Institute (ENMRI), City of Scientific Research and Technological Applications (SRTA-City), New Borg El-Arab City, Alexandria, 21934 Egypt; 2 Department of Public Works Engineering, Faculty of Engineering, Mansoura University, Mansoura, 35516 Egypt; 3 Electronic Materials Researches Department, Advanced Technology and New Materials Research Institute (ATNMRI), City of Scientific Research and Technological Applications (SRTA-City), New Borg El-Arab City, Alexandria, 21934 Egypt; 4 Environmental Engineering Department, Egypt-Japan University of Science and Technology (E-JUST), New Borg El Arab City, Alexandria, 21934 Egypt; 5 Department of Civil and Environmental Engineering, University of Maine, Orono, ME 04469 USA; 6 Chemical and Petrochemical Engineering Department, Egypt-Japan University of Science and Technology (E-JUST), New Borg El Arab City, Alexandria, 21934 Egypt; 7 Fabrication Technology Research Department, Advanced Technology and New Materials Research Institute (ATNMRI), City of Scientific Research and Technological Applications, Alexandria, Egypt

**Keywords:** Carbon-based materials, Chemical activation, Circular economy, Magnetization, Adsorbents

## Abstract

In this study, novel adsorbents were synthesized via the activation and magnetization of carbon spheres, graphene, and carbon nanotubes fabricated from plastics to improve their surface area and porosity and facilitate their separation from aqueous solutions. Fourier transform infrared spectroscopy “FTIR”, X-ray diffraction “XRD”, energy-dispersive X-ray spectroscopy “EDX”, transmission electron microscope “TEM”, and X-ray photoelectron spectroscopy “XPS” affirmed the successful activation and magnetization of the fabricated materials. Further, surface area analysis showed that the activation and magnetization enhanced the surface area. The weight loss ratio decreased from nearly 60% in the case of activated graphene to around 25% after magnetization, and the same trend was observed in the other materials confirming that magnetization improved the thermal stability of the fabricated materials. The prepared carbonaceous materials showed superparamagnetic properties according to the magnetic saturation values obtained from vibrating sample magnetometry analysis, where the magnetic saturation values were 33.77, 38.75, and 27.18 emu/g in the presence of magnetic activated carbon spheres, graphene, and carbon nanotubes, respectively. The adsorption efficiencies of methylene blue (MB) were 76.9%, 96.3%, and 74.8% in the presence of magnetic activated carbon spheres, graphene, and carbon nanotubes, respectively. This study proposes efficient adsorbents with low cost and high adsorption efficiency that can be applied on an industrial scale to remove emerging pollutants.

## Introduction

The colossal magnitude of plastic waste in the environment has negative influences on the water lifecycle and human health (Ariza-Tarazona et al. 2020). Landfills and incinerators are frequently employed to manage plastic waste (Jiang et al. 2018). However, these approaches are not suitable and cause groundwater, air, and soil contamination (Chin et al. 2022). Therefore, proper management is essential to reduce the hazards linked to plastics’ existence in the environment and control the pollution of conventional management techniques (landfilling and incineration). The conversion of plastic wastes to value-added substances such as carbonaceous materials due to their carbon-rich content is a convenient and sustainable management approach for plastics (Samy et al. 2022). Thus, plastic bottles and cups were compiled and converted to carbon-rich materials such as carbon spheres, activated carbon, and graphene.

On the other hand, industries release their effluents containing emerging pollutants such as dyes without a suitable treatment for water sources which results in the destabilization of an ecosystem (Diab et al. 2021a; Diab et al. 2021c). Methylene blue (MB) is an artificial dye regularly utilized in various industrial and laboratory applications, including as a stain in biology and medicine (Diab et al. 2021a). Despite its widespread use, MB poses potential negative impacts on the environment. One significant concern is its potential to contaminate water sources. Improper disposal or accidental releases of MB-containing wastewater from industrial processes can result in the dye entering rivers, lakes, and other aquatic ecosystems (Salama et al. 2022b). In aquatic environments, MB may persist and accumulate, potentially causing harm to aquatic organisms. Additionally, the dye’s presence can alter water quality, impacting the natural balance of ecosystems (Aziz et al. 2018; Bharti et al. 2019). Research suggests that MB may also have toxic effects on certain aquatic organisms, further emphasizing the need for proper handling and disposal practices to minimize its environmental impact. Efforts to develop and implement eco-friendlier alternatives in various applications where MB is commonly used are crucial for mitigating its negative consequences on the environment (Singh et al. 2021).

Biological treatment processes cannot degrade emerging pollutants such as MB efficiently due to their bio-mutinous nature (Samy et al. 2023). Further, chemical and physical processes such as electrocoagulation, ion exchange, coagulation, ozonation, photocatalysis, and membrane filtration are either expensive or produce secondary contaminants (Rashid et al. 2021; Salama et al. 2022a; Salama et al. 2022b; Salama et al. 2023a). Recently, researchers have focused on removing emerging pollutants via the adsorption method due to its effortless and high decontamination performance compared to the aforementioned techniques (Diab et al. 2021a; Salama et al. 2022b). However, the high cost of conventional adsorbents and their frequent regeneration as well as the difficulty of collecting adsorbent’s particles after the treatment hinder the full-scale application of this technique (Mensah et al. 2022). To overcome this issue, synthesizing adsorbents from abundant sources such as plastics can reduce the cost of the adsorption process and reinforce the scalable application of the adsorption technique. Therefore, transforming plastics into carbon-rich materials not only overcomes the environmental problems of plastics but also participates in flagging the way for the full-scale application of adsorption technology.

To improve the adsorption capacity of carbonaceous materials, these materials can be chemically activated which increases the surface area, chemical stability, reactivity, and porosity (Inyang et al. 2016; Shokry et al. 2020). Further, chemical initiation can facilitate the regeneration process (Shindhal et al. 2021). Thus, chemical activation of the prepared carbon-based materials was performed to improve the adsorption efficiency followed by the magnetization of these materials using magnetite to easily collect the adsorbent’s particles using an external magnet after water remediation (Elkady et al. 2017). Facile collection of adsorbents after treating aqueous solutions supports the successive reuse of adsorbents which reduces the treatment cost. Accordingly, the chemical activation and magnetization of carbonaceous materials derived from waste plastics represent a sustainable solution to two interconnected environmental challenges: plastic waste accumulation and water pollution. By transforming waste into a valuable resource for pollution control, this approach embodies the principles of the circular economy and contributes to the preservation of our environment for future generations.

Herein, novel carbonaceous materials such as carbon spheres, graphene, and carbon nanotubes were fabricated using plastic wastes as precursors, activated chemically, and magnetized. The materials were characterized before activation, after activation, and after magnetization using different techniques to explore their chemical composition, functional groups, morphology, chemical structure, thermal stability, surface area, and magnetic properties. Further, the prepared materials were employed for the adsorption of MB as a model pollutant to evaluate the decontamination capacity of the fabricated adsorbents, proving the environmental applicability of the prepared materials.

## Materials and methods

### Materials and reagents

Ferric chloride anhydrous (FeCl_3_, 99%), potassium hydroxide anhydrous (KOH, 99%), ferrous sulfate heptahydrate (FeSO_4_·7H_2_O, 99%), and sodium hydroxide anhydrous (NaOH, ≥97%) were supplied from Sigma Aldrich, USA. Hydrochloric acid (HCl) (36% in H_2_O) was provided by Fisher Bio-Reagents, USA. All solutions were synthesized using distilled water. Methylene blue (MB, 99.88%) was provided by Fisher Scientific and utilized as a dye pollutant without any further purification. A stock solution of MB dye was used at 1000 ppm via disassociation of 1 g of MB powder in 1 l of distilled water (DW).

### Chemical modification and activation of the high-carbon value materials

In the chemical activation process, activation time, the chemical agent influence, temperature, impregnation ratio, and activation media (under atmospheric environments) were investigated and optimized. Alkaline groups are strong potential activation agents; hence, potassium hydroxide (KOH) was selected to perform the modification process. The char, graphene, and CNT used as carbon sources for the chemical activation and magnetization were prepared from plastic wastes as designated in our previous study (Mensah et al. 2022; Salama et al. 2024).

#### Activation of plastic-char

One gram of the previously prepared char was placed in a semi-closed jar with freshly prepared KOH 40% at the optimized ratio of 1:4, respectively (Salama et al. 2024). The homogeneous mixture was positioned into a muffle furnace at 700 °C for 2 h and allowed to cool overnight. The resulting char was washed twice using distilled water followed by washing using HCL and finally with distilled water again. The final produced material was dried using an oven for 12 h at 80 °C.

#### Activation of graphene

One gram of the previously prepared graphene (Salama et al. 2024) was soaked overnight in an aqueous 40% KOH solution until maximum absorption. The extra solution was carefully removed, and the mixture was placed into a tightly closed jar in a muffle furnace for 2 h at 500 °C. When the jar reached room temperature, the prepared sample was removed and cleaned using DW and HCL till a neutral pH was recorded and left to dry in an oven at 80 °C and then ground into fine particles.

#### Activation of carbon nanotubes

The purified carbon nanotubes (CNTs) were mixed properly in a mortar with KOH solution in a ratio of 1:5 until they reached the consistency of a precipitate. The mixture was positioned in a stainless jar and flushed with nitrogen then tightly sealed. The sealed jar was introduced to a muffle for 2 h at 700 °C then left to cool overnight.

#### Magnetization of the prepared high-carbon value materials

Firstly, 4 g of FeCl_3_ was liquefied in 200 ml of DW, and then placed onto a hotplate under continuous stirring till completely dissolved. Two grams of FeSO4·7H2O was dissolved in 100 ml DW and added to the mixture. When the temperature reached 70 °C, 1 g of the carbon-based materials (plastic char, graphene, or carbon nanotubes) were placed in the solution then 50 ml of 5 M NaOH was slowly dropped into the abovementioned mixture. The final solution was further stirred for 30 min at 70 °C and left to cool. Using a centrifuge, the mixture was washed with DW followed by ethanol and DW again. Finally, the washed powder was dried using an oven overnight.

### Characterization of the prepared carbonous materials

The magnetization and activation of the fabricated materials were affirmed using different characterization techniques. Fourier transform infrared (Bruker Bremen, Germany) was utilized to examine the practical groups of the prepared carbon materials. Moreover, X-ray diffraction (Schimadzu-7000, Shimadzu Co., Kyoto, Japan) was employed via an X-ray diffractometer (CuK α1 radiation, *λ* = 1.54 Å) at 40.0 mA, 40.0 kV and 2θ from 20 to 80° during 30 min to investigate the crystallinity of the fabricated materials. Moreover, a transmission electron microscope (JEOL, JEM-2100, Japan) tandem by energy dispersive X-ray spectroscopy was used to investigate the morphological and chemical composition of the fabricated carbonous materials. Furthermore, X-ray photoelectron spectroscopy (Thermo Scientific, USA) was utilized to explore the chemical statuses of the prepared materials. The Brunauer–Emmett–Teller (Beckman Coulter SA3100, USA) analyzer was used at 77 K to determine the surface area by detection of the nitrogen adsorption/desorption isotherms. The fabricated carbonous materials were degassed beforehand analysis under vacuum for 12 h at 25 °C. The thermogravimetric analyzer (Shimadzu TGA-50 instrument) was utilized to assess the thermal stability of the synthesized carbonic materials. Finally, the magnetic features of the fabricated carbonous composites were characterized via the vibrating sample magnetometer (Dexing 250, USA) at 25 °C.

### Adsorption test

The adsorption performance of the fabricated carbonaceous materials was assessed using 10 mg/L of MB as an adsorbate at pH = 7, contact time of 120 min, carbonous materials dose of 1 g/L, and room temperature. The decontamination experiments were performed in a 250-mL beaker, and the volume of the polluted solution was 200 mL. A hotplate stirrer was used to mix the solution at 150 rpm. Samples were withdrawn and centrifuged before analysis. The concentration of MB dye was measured by quantifying the absorbance at 665 nm using a UV-Vis spectrophotometer (V-630, Shimadzu, Japan). The MB removal efficiency was calculated using Eq. (1).

1 $$R\%=\frac{\left({C}_0-{C}_{\textrm{t}}\right)}{C_0}\times 100\%$$

where *C*_0_ is the initial concentration and *C*_t_ is the concentration after adsorption at time, *t*.

## Results and discussion

### Characterization of the prepared carbon-based materials

#### Characterization of char

**Fig. 1:**
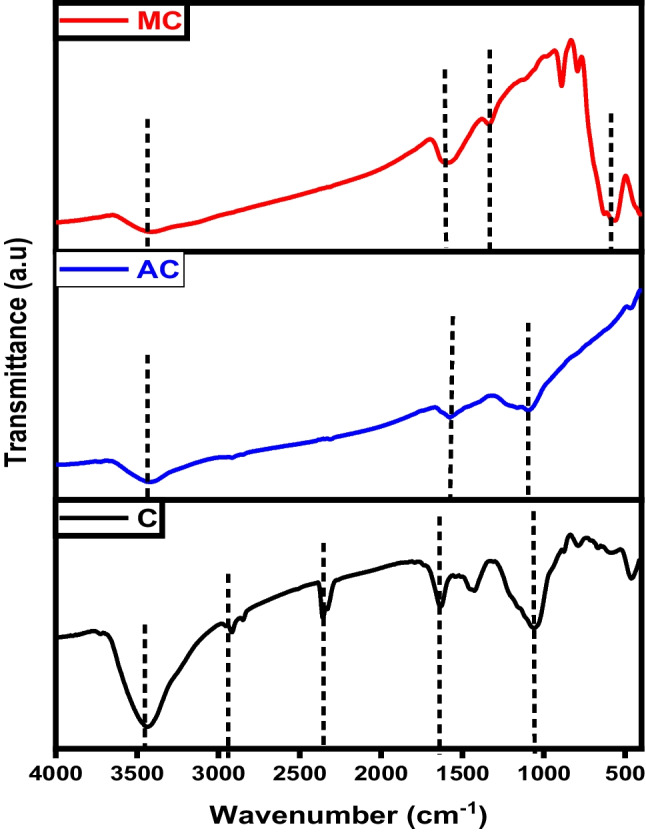
FTIR analysis of char (C), activated carbon (AC), and magnetic carbon (MC)

Figure 1 displays the FTIR spectra of char, activated carbon, and magnetic carbon. In all three spectra, a broad band around 3400 cm^−1^ is observed, indicating the –OH stretching vibration of hydroxyl functional groups (Gotore et al. 2022). Additionally, smaller bands between 1150 and 1680 cm^−1^ are identified, corresponding to C=C stretch vibrations of aromatic ring structures and carbonyl groups stretching, typical for carbonaceous materials (Salama et al. 2024; Shokry et al. 2020). These bands may appear as a result of the breakdown of C–H bonds at higher activation temperatures into a more stable formula of aromatic C=C bonds. They may also stem from C=O groups coupled with aromatic rings. Moreover, in the magnetic composite spectrum, there is a prominent band at 550 cm^−1^, indicating Fe-O bond vibrations at octahedral and tetrahedral positions, which demonstrates the existence of Fe_3_O_4_ particles within the magnetic structure (Salama et al. 2024).

**Fig. 2:**
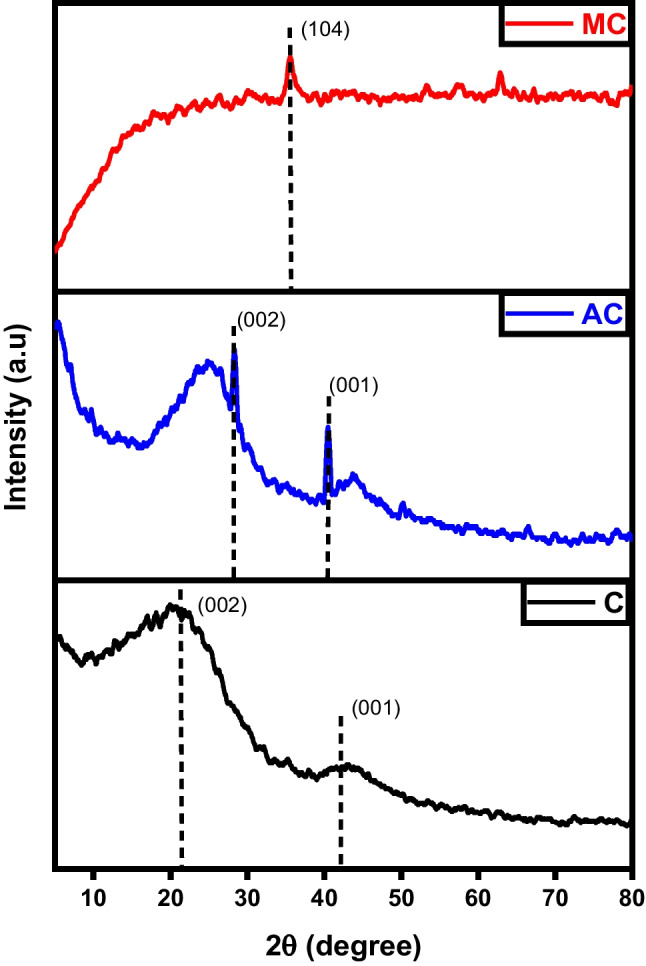
XRD analysis of char (C), activated carbon (AC), and magnetic carbon (MC)

The XRD diffractograms depicted in Fig. 2 revealed that the carbon char initially exhibited an amorphous structure before modification, as evidenced by the reflection of the plane (002), a characteristic trait of non-crystalline structures like activated carbon. After activation, there was a discernible transformation as exfoliated graphite-like crystals began to form, as evidenced by the emergence of peaks at 2θ=29° and 43° (Salama et al. 2024). These significant results reveal the success of the activation process using KOH which produced a high crystallinity activated carbon with improved characteristics. On the other hand, the diffractograms of magnetic carbon illustrate a drastic change in peak analysis. The usual peaks corresponding to the (002) and (001) planes are normalized, and an intense peak assigned to Fe_3_O_4_ at 2θ 38.3° emerged which refers to the crystalline nature of Fe_3_O_4_ represented by the (104) plane (Albdiry and Al-Nayili 2023).

**Fig. 3:**
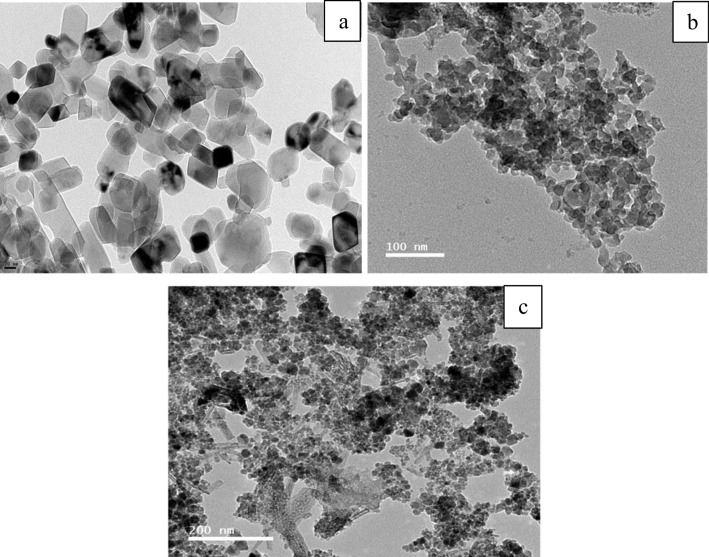
TEM images of **a** char, **b** activated carbon, and **c** magnetic carbon

As depicted in Fig. 3a, the carbon samples produced exhibited irregular shapes, falling within the nano-scale range in size when compared to the images of activated carbon. In Fig. 3b, the surface porosities became more pronounced, and the hierarchical structures comprising circular-shaped particles were distinctly detected. During the magnetization process, the iron complex anion which is produced by some iron-chloride aids the graphitization of the activated char/carbon. The iron-based complexes lose their halogen atoms and turn into iron oxides which can be noticed as random rod-shaped particles as seen in Fig. 3c. The uniformity of shape and size could be maintained by proceeding with the carbonization under relatively mild conditions.

**Fig. 4:**
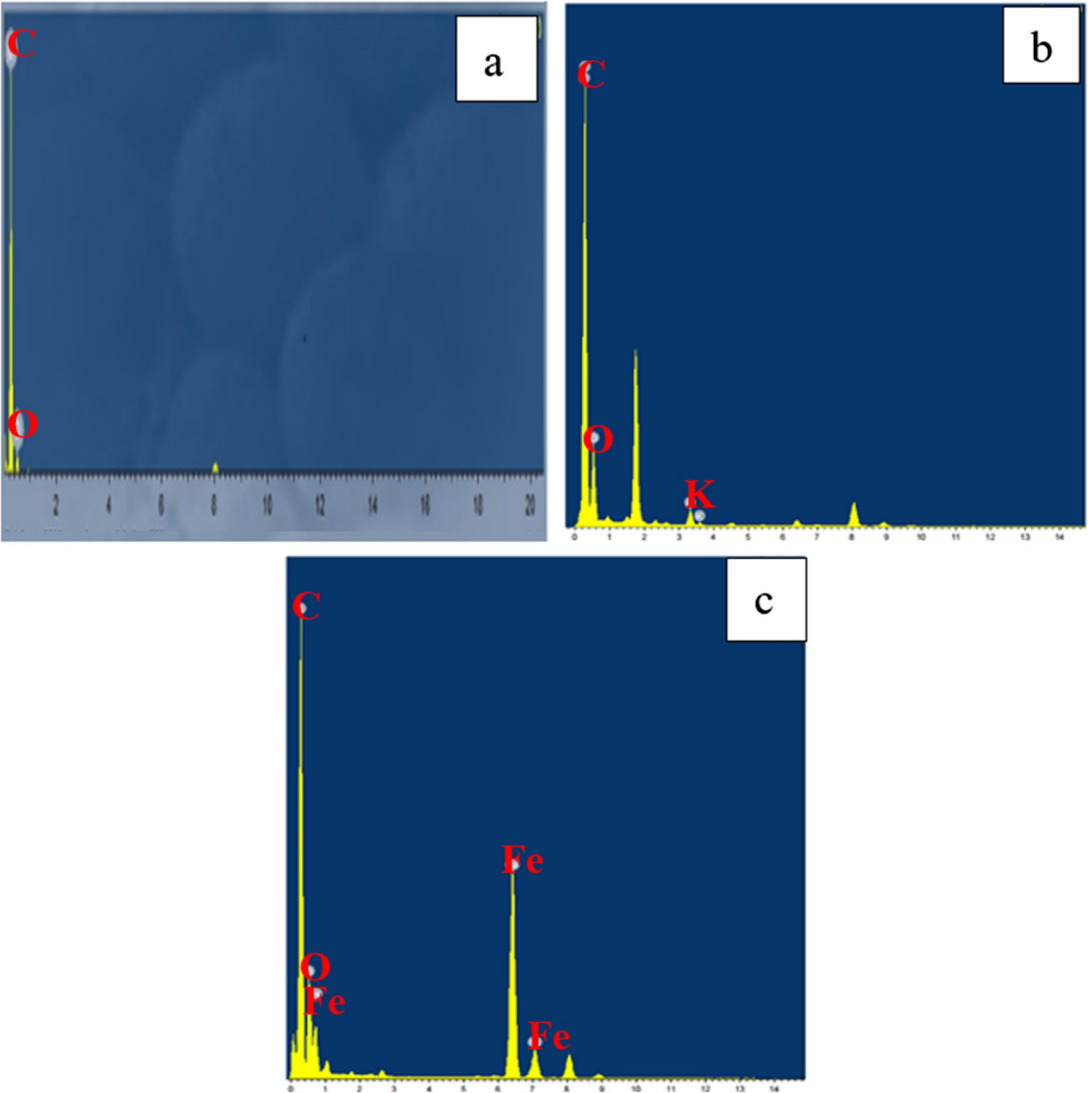
EDX analysis of **a** char, **b** activated carbon, and **c** magnetic carbon

The EDX analysis revealed the presence of carbon (C) and oxygen (O) in the fabricated char, as illustrated in Fig. 4a. The quantification of the peaks indicated atomic percentages of approximately 97.98% for carbon and 2.02% for oxygen. This high carbon content, coupled with the absence of other contaminants, suggests that the pyrolysis process was successful (Hsu et al. 2023). The activated carbon shown in Fig. 4b contains a slightly higher percentage of O (7.9%) with traces of K (1.1%) that are remaining from the alkaline activation process. In the same contest, the magnetization process had a notable effect on the elemental analysis where iron accounted for 24.12% of the final yield (Fig. 4c). These results are in agreement with the data reported in previous literature (Mensah et al. 2022).

**Fig. 5:**
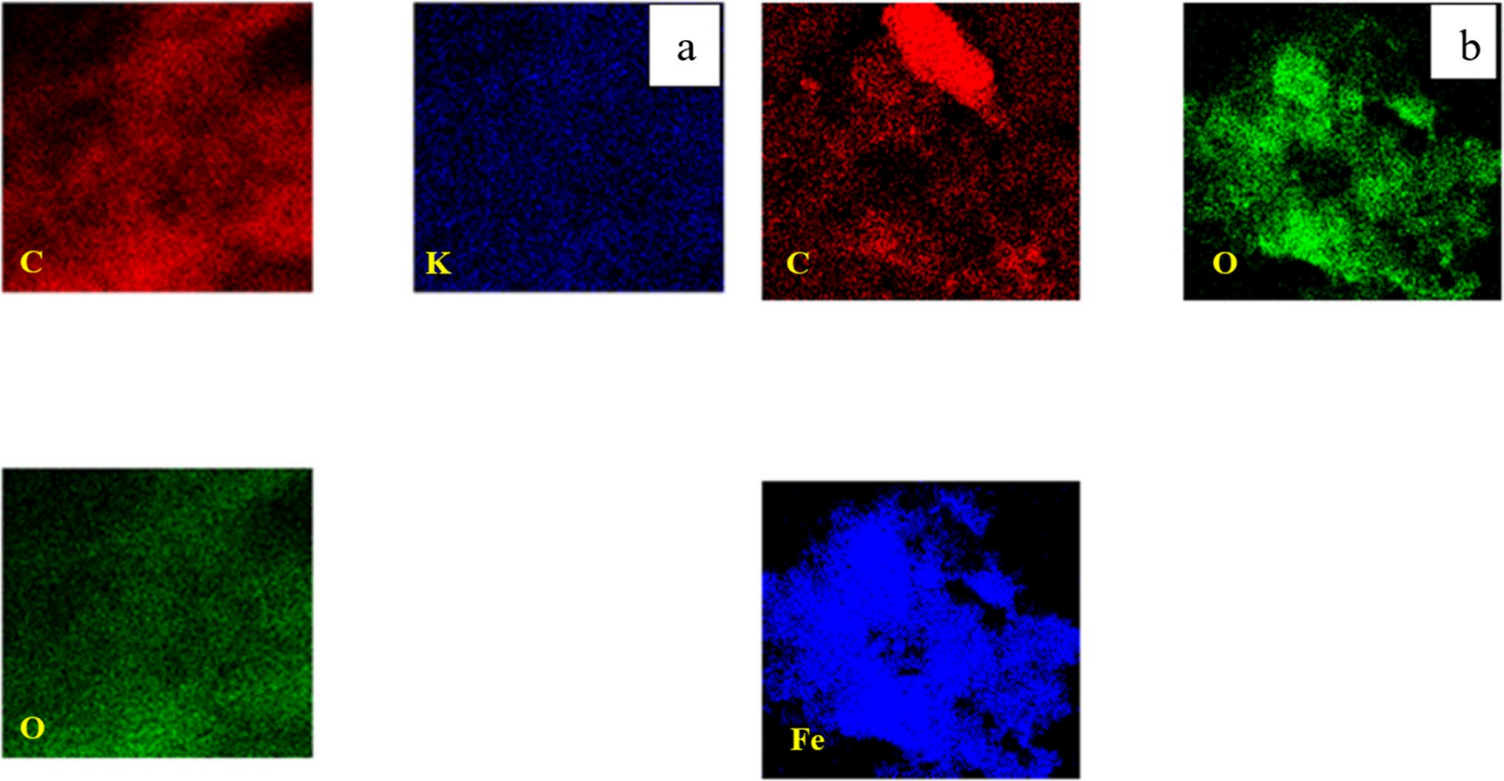
Mapping of **a** activated carbon and **b** magnetic carbon

EDX mapping was conducted on both activated and magnetic carbon. The images taken in Fig. 5 show an equal distribution of carbon, and oxygen along with potassium (activation agent) and iron (magnetizing agent), which suggests that the activation and magnetization of the carbon-based materials occurred homogeneously (Shokry et al. 2020).

**Fig. 6:**
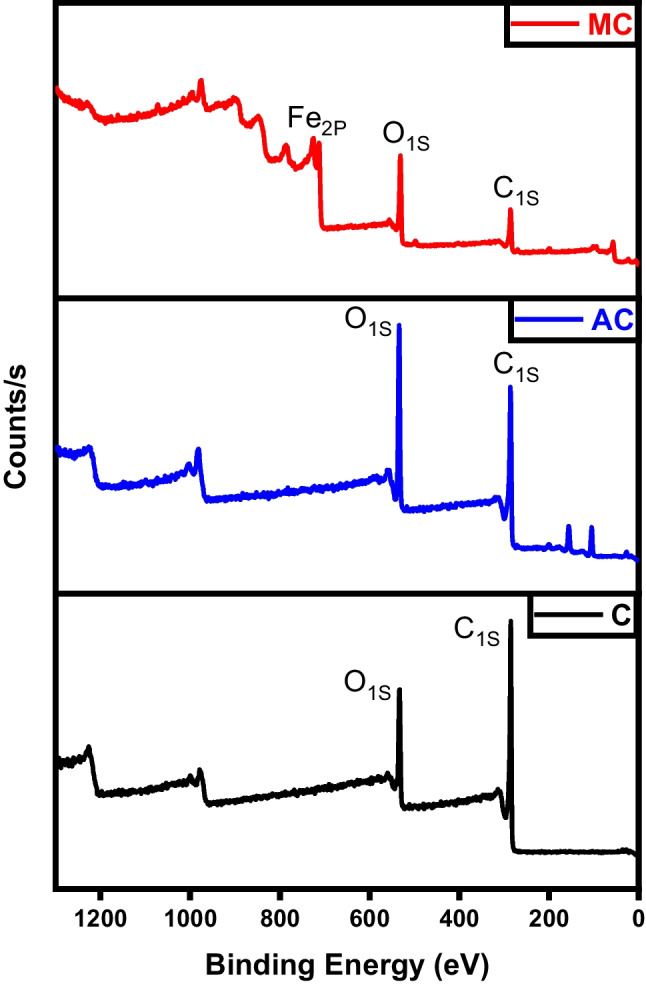
XPS analysis of char (C), activated carbon (AC), and magnetic carbon (MC)

The XPS investigation of the char, activated carbon, and magnetic carbon is depicted in Fig. 6. This test aimed to identify the surface species composition, their binding energies, and oxidation states. The primary C_1S_ peak observed in both char and activated carbon appeared at a binding energy of 284.6 eV, corresponding to C–C bonding (Salama et al. 2024). On the other hand, three distinctive iron peaks besides carbon and oxygen are located in the magnetic char analysis. The position of the Fe (2p_3/2_) and Fe (2p_1/2_) peaks were noticeable at 712.39, 726.9, and 733.77 eV, respectively. The values observed are consistent with those reported for γ-Fe_2_O. These results refer to the construction of γ-Fe_2_O_3_ in the magnetic fabricated samples (Salama et al. 2022c).

**Fig. 7:**
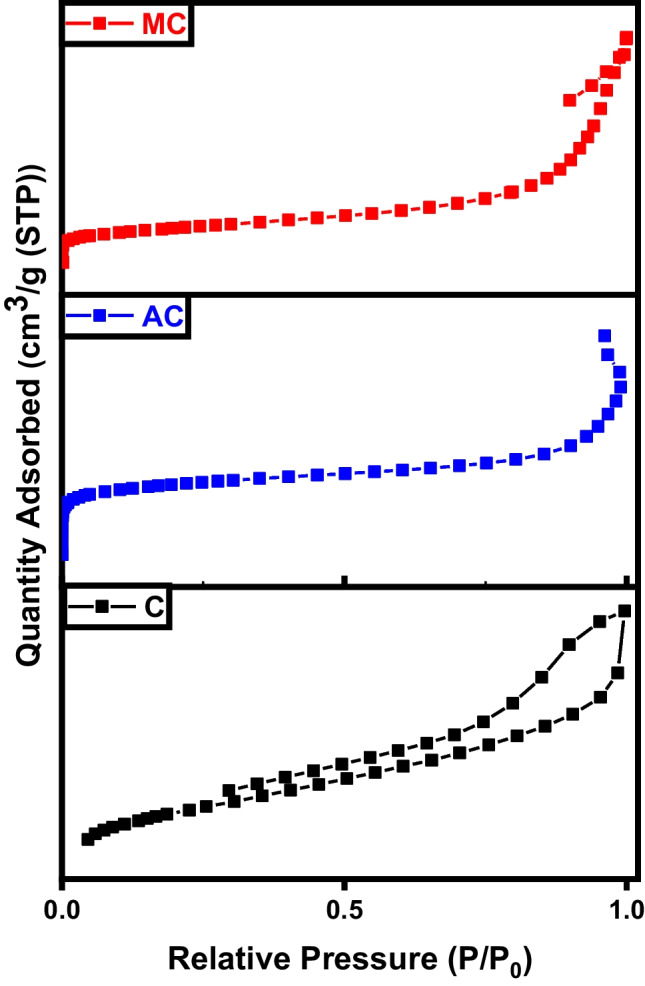
BET analysis of char (C), activated carbon (AC), and magnetic carbon (MC)

As shown in Fig. 7, the N_2_ adsorption/desorption isotherms of char, activated, and magnetic carbon prepared. The adsorption isotherms observed for the produced samples exhibit a combination of type I and type IV isotherms according to the IUPAC classification, indicating the presence of both mesopores and micropores within the carbonic structure. At low pressures, there is an increase in the amount of N_2_ adsorbed, indicating the higher microporosity of activated carbon. Moreover, type I isotherm is usually associated with microporous samples characterized by a relatively small external surface area. However, in activated carbon, some mesopores are present, as evidenced by a slight increase in N_2_ adsorption at higher relative pressures after filling the micropores (Yang et al. 2023). The hysteresis loops observed in all desorption isotherms indicate the presence of slit-shaped micropores or mesopores in the structure of these samples. The specific surface area, total pore volume, and average pore diameter presented in Table 1 illustrate the enhancement in porosity and surface area of the char after activation and magnetization (Erdogan et al. 2023).

**Table 1:** BET data of C char sample

Sample	Specific surface area (m^2^ g^−1^)	Total pore volume (cm^3^ g^−1^)	Average pore diameter (nm)
C	4.1	0.283	9.5
AC	12	0.048	46.9
MC	79	0.3622	19.9

**Fig. 8:**
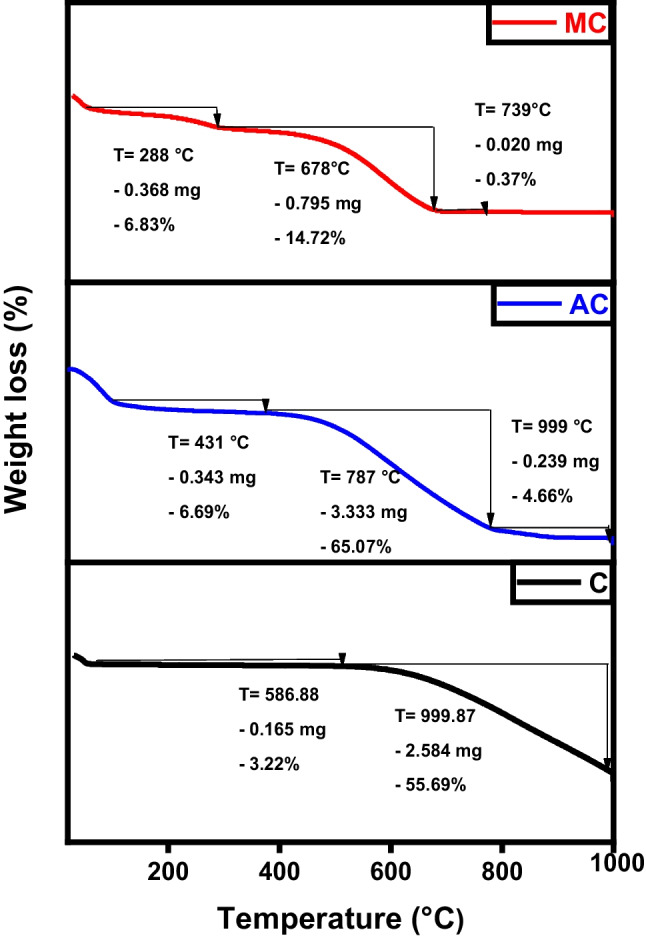
TGA analysis of char (C), activated carbon (AC), and magnetic carbon (MC)

Thermogravimetric analysis (TGA) assesses the thermal stability of the fabricated samples, as demonstrated in Fig. 8 for raw char, activated, and magnetic materials at several heating rates in the presence of a nitrogen atmosphere. Typically, four stages of thermal decomposition are observed: between 50 and 130 °C (associated with water evolution), 130–220 °C (involving degradation and evaporation of volatile compounds), 220–460 °C (depolymerization pyrolysis), and >460 °C (carbonic decomposition). For all produced samples, a slight loss of the total weight occurs at the very early stage due to water evaporation till a temperature of 150 °C. The raw char, the sample existence of a mild weight loss attributed to the desorption of the physiosorbed water in conjunction with the rigidly bound water. This phase is followed by a rapid deterioration starting at 586 °C where most of the sample (55.6% weight) is decomposed due to the breakage of internal polymeric chains. The activated carbon sample experiences a similar behavior where it loses the majority of its weight between 431 and 787 °C. Finally, magnetic carbon is highly resistant to heat decomposition as the presence of ferric atoms greatly enhances thermal stability (Diab et al. 2021b; Salama et al. 2022b).

**Fig. 9:**
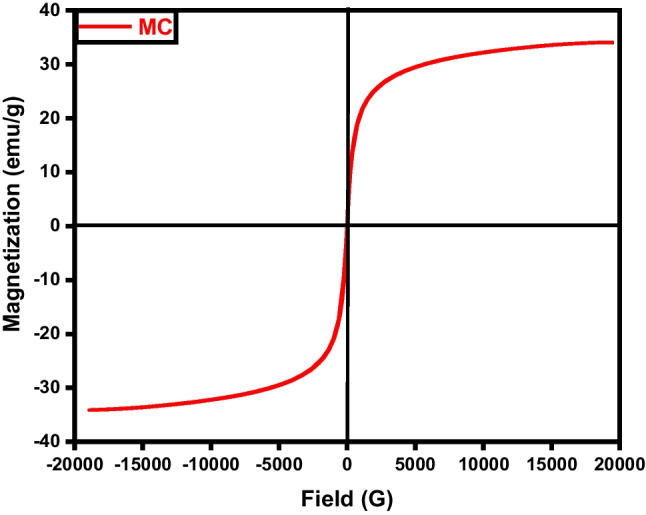
VSM analysis of magnetic carbon (MC)

The samples’ magnetic field and strength were assessed through the utilization of a vibrating sample magnetometer (VSM). Where, the magnetic properties of the iron/carbon composite are represented by an S-like loop (Fig. 9) implying that the sample exhibits a superparamagnetic characteristic, which is typically observed in small ferromagnetic or ferrimagnetic nanoparticles, where there’s a random fluctuation in magnetization direction due to temperature effects (Elkady et al. 2020; Salama et al. 2023b). The saturation magnetization (Ms) was measured to be 33.77 and −34.07emu/g, respectively.

#### Characterization of graphene

**Fig. 10:**
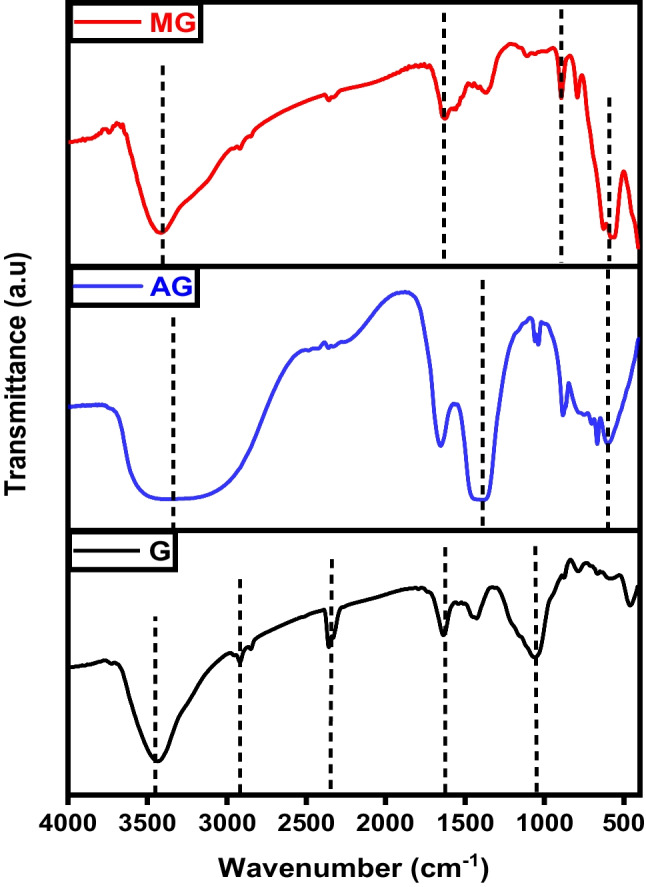
FTIR analysis of graphene (G), activated graphene (AG), and magnetic graphene (MG)

The FTIR spectra of graphene, KOH-activated graphene, and magnetic graphene are illustrated in Fig. 10. All three plots share the presence of the signature O-H band at about 3400 cm^−1^ of hydroxyl or carboxyl groups (Gotore et al. 2022). For graphene spectra, the robust peak located around 1572–1580 cm^−1^ is assigned to the vibrations of the skeletal C=C in the aromatic structure. It also illustrates C=C=O stretching vibrations at 2918.3 cm^−1^ and 2358.33 cm^−1^ (Salama et al. 2024). Carboxylates or ketones C=O stretching appeared at 1636.17 cm^−1^ and C-O stretching vibrations at 1060.64 cm^−1^ (Gotore et al. 2022). The presence of oxygen-containing groups in graphene was notably reduced, and some of these bonds vanished following chemical activation. Additionally, a new band emerged at 1384 cm^−1^, attributed to -OH bending. The broad peaks in the case of the activated graphene (AG) are due to the introduction of the OH group as a result of the activation by KOH (Gotore et al. 2022; Mensah et al. 2022). For magnetic graphene, the characteristic peaks at ~ 580 cm^−1^ and ~ 628 cm^−1^ belong to Fe–O stretching vibrations, suggesting that the magnetic composite was successfully synthesized (Pavía et al. 2023).

**Fig. 11:**
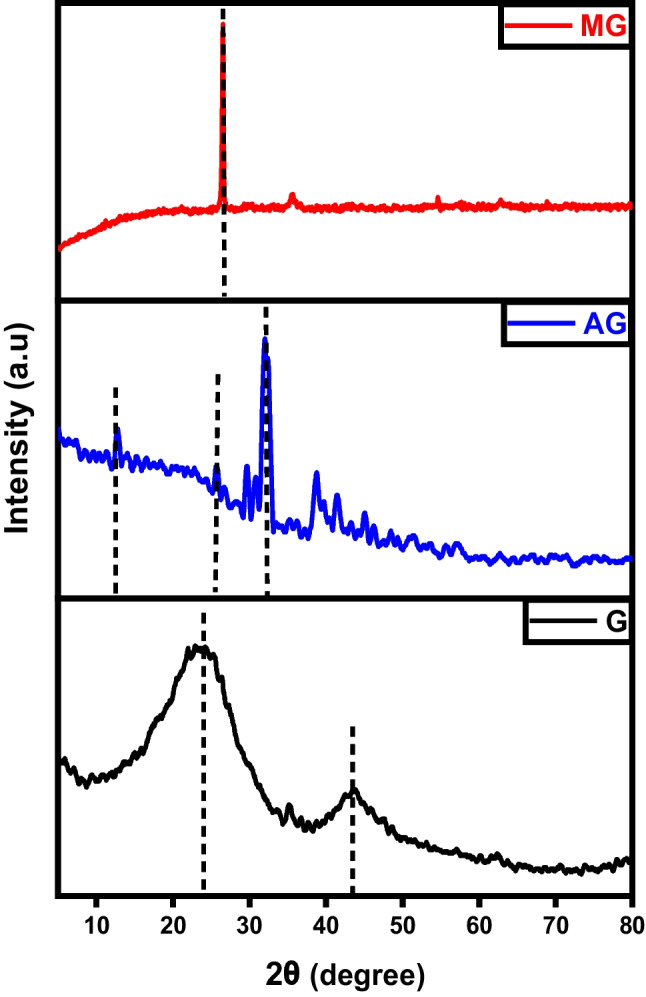
XRD analysis of graphene (G), activated graphene (AG), and magnetic graphene (MG)

Figure 11 characterizes the X-ray diffraction patterns of graphene, activated, and magnetic graphene. A distinct diffraction peak observed at approximately 2θ = 25° can be attributed to the (002) crystal plane of graphite, suggesting the well-ordered structure of graphene layers with moderate spacing. For graphene, the characteristic diffraction peak of 2θ = 43.74° assigned to the 101-crystal plane indicates that there is an increasing trend in the crystalline morphology (Habib et al. 2022). As for activated graphene, the presence of a characteristic diffraction peak at 2θ = 12.2° corresponding to the (001) plane confirms the successful oxidation of graphene and the construction of oxygenated groups for example epoxide, hydroxyl, carbonyl, and carboxyl groups in graphite oxide (Harres et al. 2023).

**Fig. 12:**
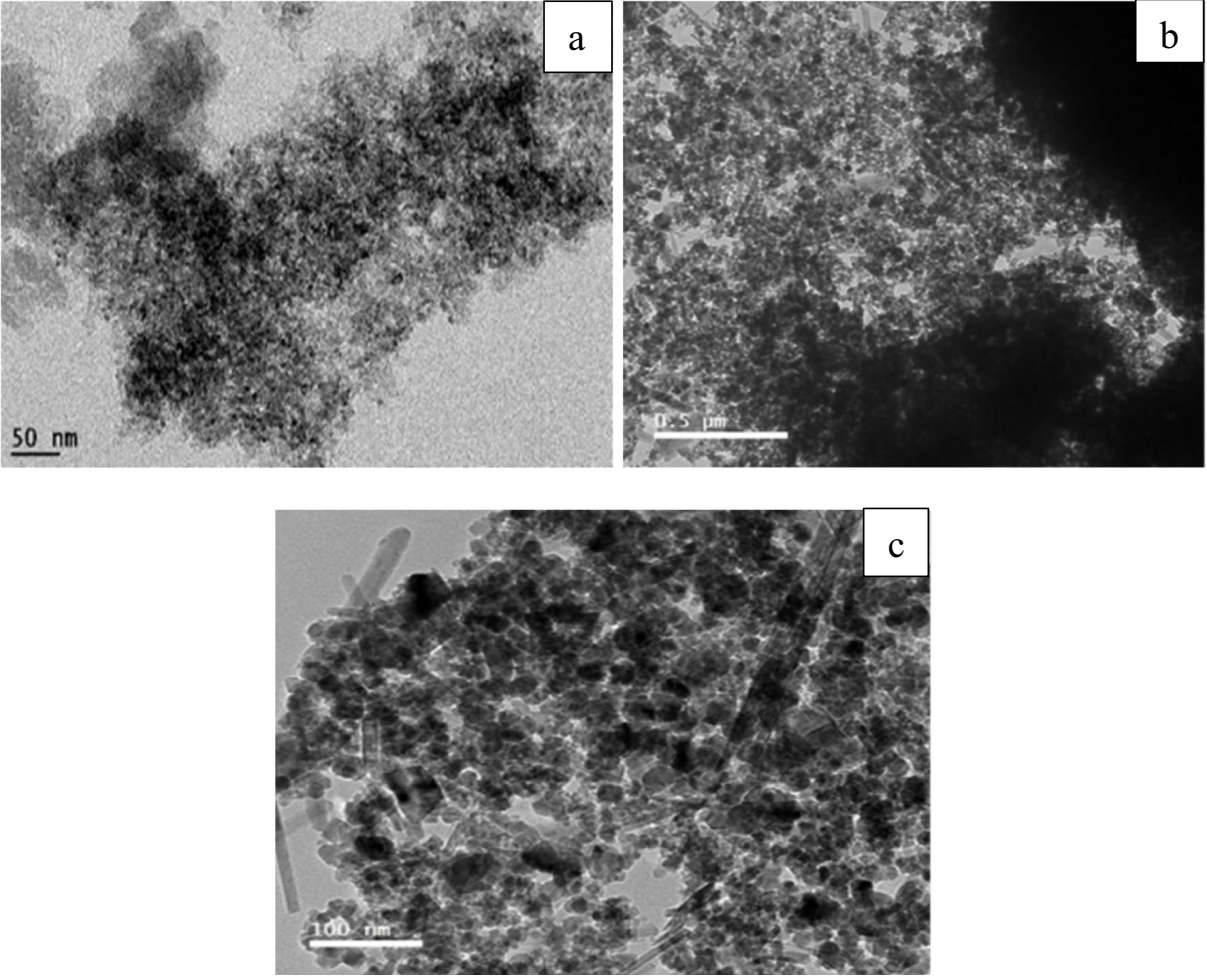
TEM images of **a** graphene, **b** activated graphene, and **c** magnetic graphene

The morphology of the three graphitic structures was inspected using TEM. As shown, in all samples’ crystallinity was not affected by the process conditions. The microporosity of the activated graphene remained intact even after activation as seen in Fig. 12b. Figure 12c shows that the iron oxide rod-shaped particles are resolutely connected to the graphene particles, which assistances to prevent iron agglomeration and allows a well-distribution of the oxide particles on the graphene.

**Fig. 13:**
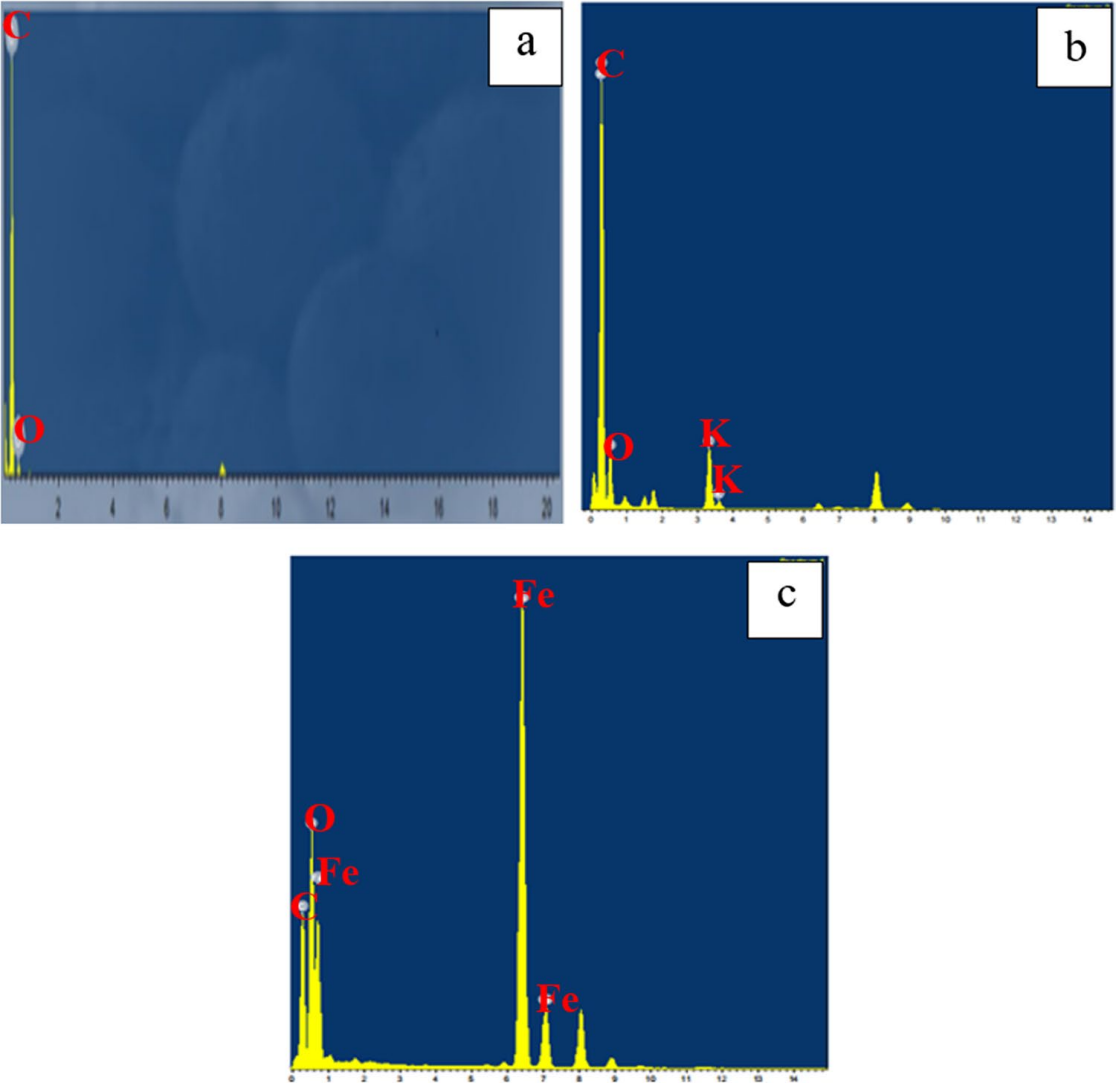
EDX images of **a** graphene, **b** activated graphene, and **c** magnetic graphene

Figure 13 displays the difference between the EDX patterns of graphene, activated, and magnetic graphene. As expected, a new peak corresponding to iron oxide appeared in the case of magnetic graphene conjugated with the formation of the magnetic nanocomposite. Moreover, the atomic percentage of carbon decreased from 97.98% in pure graphite to less than 50%. For activated graphene, the carbon remained at 90.16% and also traces of the activation agent (KOH) appeared in the spectra (Habib et al. 2022).

**Fig. 14:**
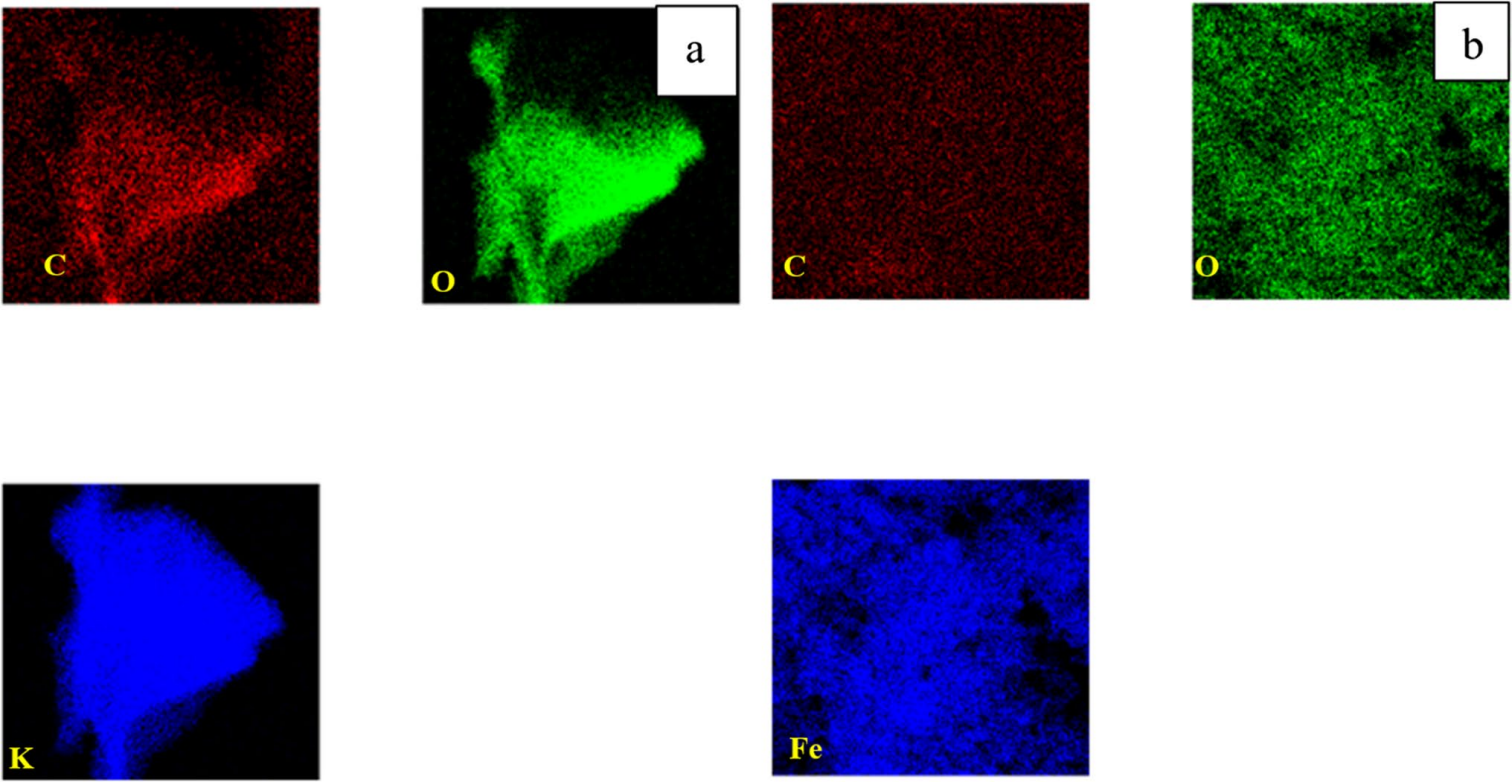
Mapping of **a** activated graphene, and **b** magnetic graphene

The elemental mapping of activated and magnetic graphene indicates the existence of elements carbon and oxygen with uniform distribution along both samples (Fig. 14). Traces of potassium could be located in the activated sample while a proper amount of iron was found in the magnetic compound (Ayub et al. 2023).

**Fig. 15:**
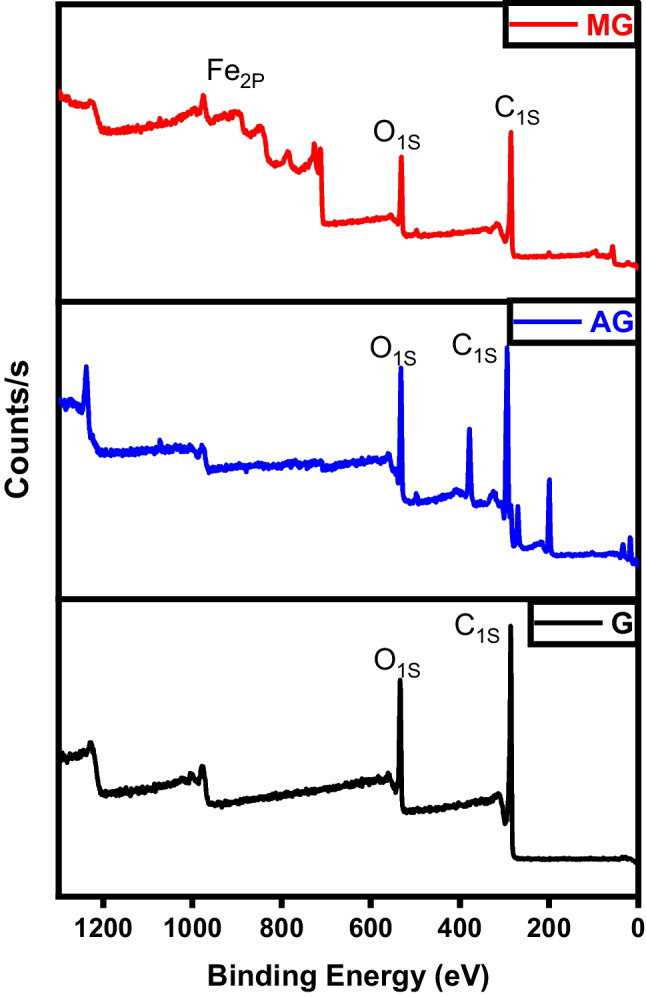
XPS analysis of graphene (G), activated graphene (AG), and magnetic graphene (MG)

As represented in Fig. 15, high-resolution spectra were acquired for the three structures prepared. The deconvolution of the C_1S_ pattern is primarily utilized to assess the oxidation or reduction degree that the graphene was subject to chemical modification. The three deconvoluted patterns of C_1S_ illustrated at binding energies of 285.95 eV (graphene), 285.47 eV (activated graphene), and 294.15 eV (magnetic graphene) serve to characterize the chemical state (oxidation level) of the graphene-based structures (Ojeda et al. 2023). Reduction of graphene by KOH led to a reduction in the presence of oxygen-bound carbons (C-OH/C=O), accompanied by an increase in carbon hybridization. Magnetic graphene exhibited patterns of Fe 2P_3/2_ and Fe 2P_1/2_ at 712.42 and 754.79 eV, respectively, corresponding to the energy level of the 2p orbital. It is concluded that iron was successfully impeded within the graphitic structure (Hou et al. 2016).

**Fig. 16:**
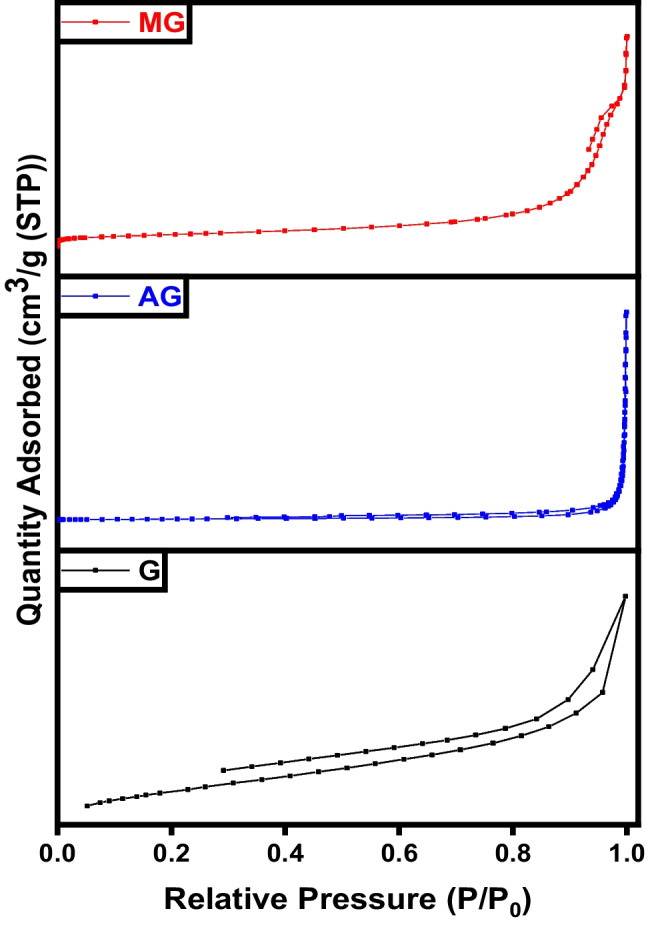
BET analysis of graphene (G), activated graphene (AG), and magnetic graphene (MG)

Figure 16 represents the hysteresis loop shapes of the graphitic structures. The area of the hysteresis loops serves to prove that the number of mesopores marginally rises with the enhancement oxidation of the sample. As per the IUPAC classification, all nitrogen adsorption-desorption isotherms for the obtained materials exhibit type IV isotherms, indicating the presence of mesopores. Both meso- and micropores were observed in the graphene sample, which displayed type IV isotherms. The mesoporosity in graphene is attributed to the high temperature of the pyrolysis process. The isotherm of activated and magnetic graphene indicates an H_3_ hysteresis loop (Albaik et al. 2023; Salama et al. 2022a). This loop begins at a relative pressure range of 0.8–1, indicating the occurrence of capillary condensation phenomena, characteristic of mesoporous materials. Hysteresis occurs when the adsorption and desorption curves do not overlap, primarily associated with the capillary condensation phenomenon that occurs in mesoporous structures during the desorption process. In this scenario, more hysteresis loops were observed for the activated and magnetic graphene, elucidating the enhanced formation of mesopores compared to pure graphene (Salama et al. 2023a; Salama et al. 2024). Table 2 exhibits the improvement in surface area and porosity of the graphene after activation and magnetization.

**Table 2:** BET analysis of graphene-based samples

Sample	Specific surface area (m^2^ g^**−**1^)	Average pore diameter (nm)	Total pore volume (cm^3^ g^**−**1^)
G	548	1.6	0.214
AG	631	3.2	0.504
MG	404	9.2	0.473

**Fig. 17:**
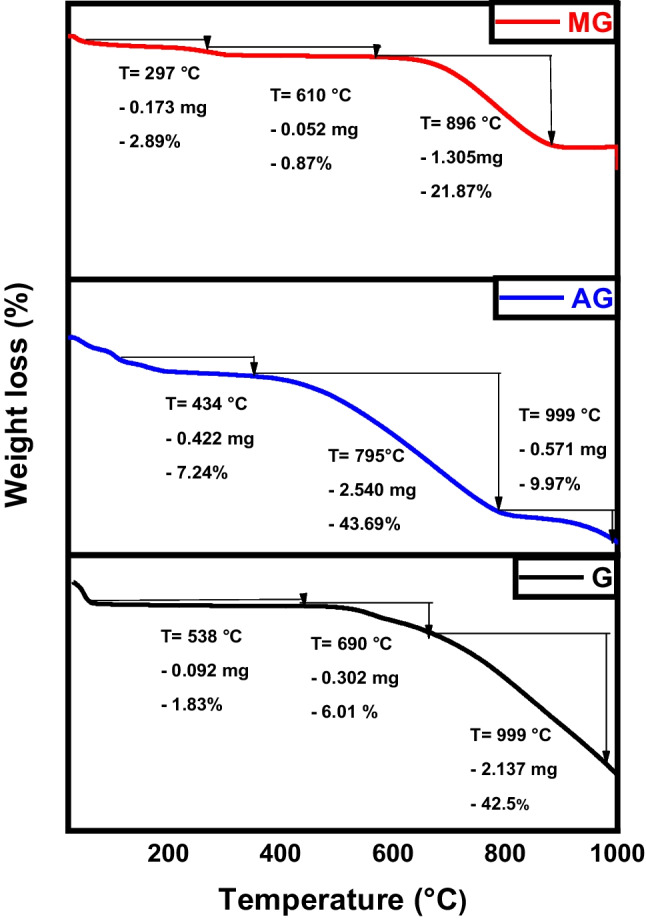
TGA analysis of graphene (G), activated graphene (AG), and magnetic graphene (MG)

The prepared graphene, activated, and magnetic graphene were exposed to thermal degradation up to 1000 °C to evaluate their thermal characteristics. Figure 17 signifies the thermal peaks of the graphene-derivatives. The lower temperature required to overcome sp^2^ hybridized carbon bonds in graphene and activated graphene compared to magnetic graphene is observed from the plots (Bagheri and Baharfar 2023). Magnetic graphene has a higher decomposing temperature as it requires a bigger quantity of heat energy to decompose the carbon/iron atomic bonds present in the hexagonal carbon structure. Due to a strong 3D carbon framework and involving of a great number of iron/graphene-stacked films detained by extra van der Waals forces, the magnetic sample lost about 25% only of its total weight during the test compared to more than 60% weight for the graphene and activated graphene samples (Salama et al. 2023b).

**Fig. 18:**
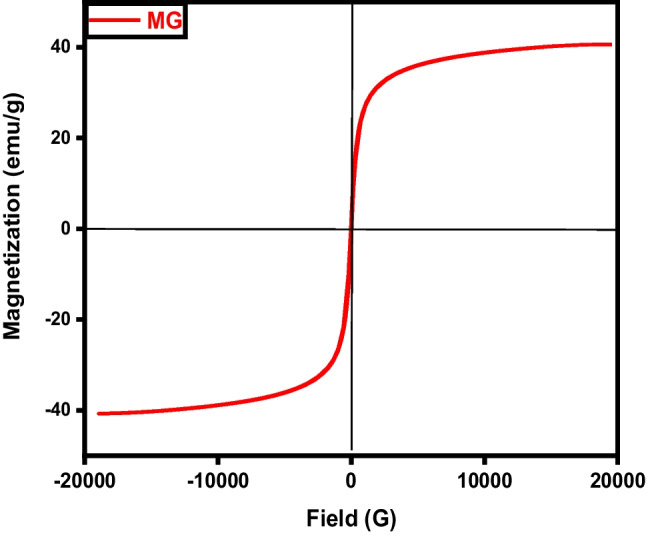
VSM analysis of magnetic graphene (MG)

The magnetic hysteresis loop exposed in Fig. 18 obviously indicates the ferromagnetism of the fabricated magnetic graphene sample (Salama et al. 2023b; Shokry et al. 2020). The saturation magnetizations (Ms) and coercive forces (Hc) can be derived from the magnetic hysteresis loop, with Ms being measured at 38.75 emu/g.

#### Characterization of carbon nanotubes

**Fig. 19:**
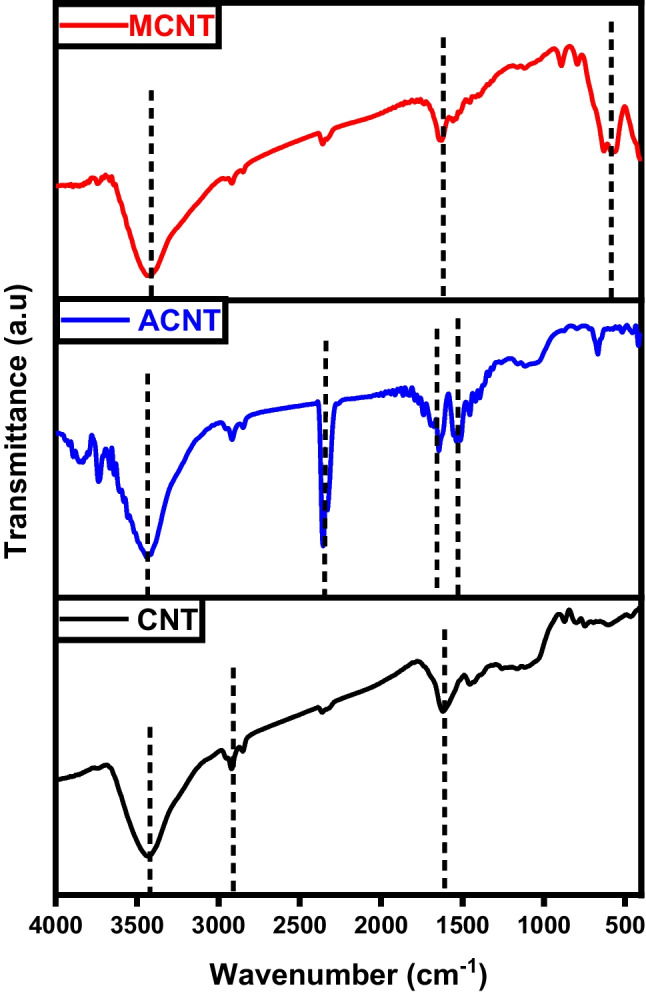
FTIR analysis of carbon nanotubes (CNTs), activated carbon nanotubes (ACNTs), and magnetic carbon nanotubes (MCNTs)

FTIR spectra of pure CNTs, activated CNTs, iron-equipped CNTs materials are illustrated in Fig. 19. The absorption bands observed around 3440 cm^–1^ in the spectrum of CNTs are attributed to the stretching and bending vibrations of O–H in hydroxyl groups. These hydroxyl groups may exist as isolated O–H moieties or may be due to adsorbed water molecules. The smaller peaks at 2920 and 2850 cm^–1^ correspond to the stretching vibration of the C–H bond. The infrared spectra peak at 1620 cm^–1^ is attributed to the stretching vibration of carboxyl groups C=O (Pereira et al. 2023). The band at 1455 cm^–1^ is indicative of C=C bonds, and the shift in this band from 1600 cm^–1^ indicates changes in the carbon structure upon carboxylation. For activated CNTs, the band observed around 2900 cm^−1^ corresponds to the sp^3^ C–H stretching vibration in aliphatic and aromatic structures. The sharp asymmetric peaks around 1700 cm^−1^ can be attributed to the C=O stretching vibrations. As the activation temperature increases in both chemical and physical activation processes, the features associated with carbon-oxygen bonds become less pronounced. The bands assigned to magnetic CNTs at 3416 and 1627 cm^−1^ refer to the stretching and bending vibrations of the O–H and C=O groups, respectively. Furthermore, the peak at 580 cm^−1^ represented the stretching vibration of Fe–O of Fe_3_O_4_ (Salama et al. 2024).

**Fig. 20:**
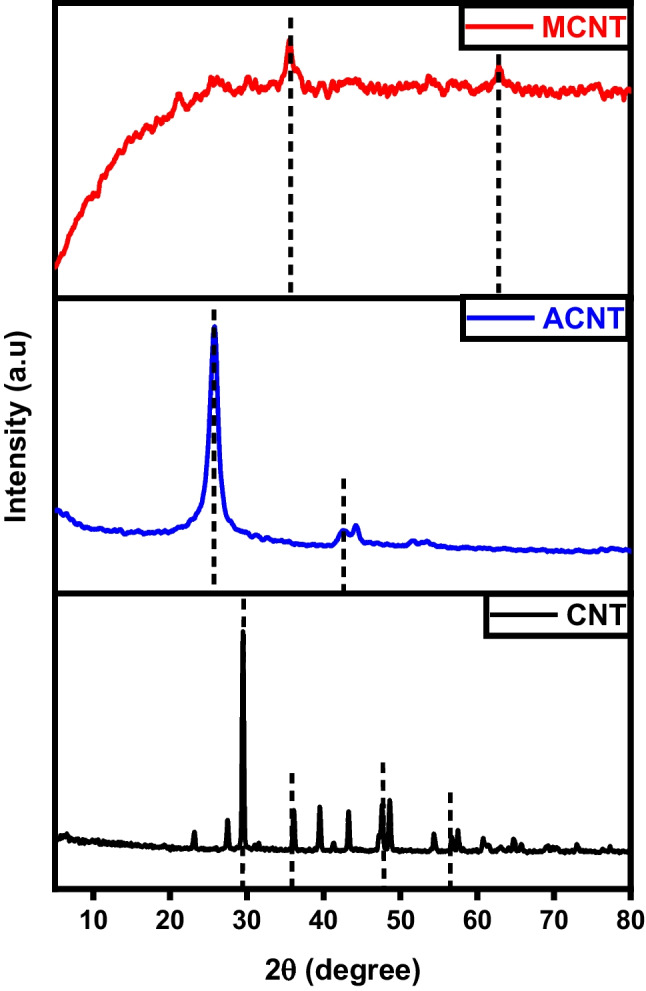
XRD analysis of carbon nanotubes (CNTs), activated carbon nanotubes (ACNTs), and magnetic carbon nanotubes (MCNTs)

The powder X-ray diffraction (XRD) analysis reveals the crystalline structure, as depicted in Fig. 20. A sharp and intense peak observed at 2θ = 29.4° matches the (002) plane within a tetragonal structured carbon atom which indicates a high degree of crystallinity in the carbon structure. Despite the sharp peak at the (002) plane, the resultant carbon material may exhibit non-crystalline characteristics with a periodic structure, leading to distinct X-ray diffraction peaks. In the case of activated CNTs, the diffraction peak at 2θ = 26° can confidently be identified as the (002) reflection, similar to pure CNTs. Additionally, other minor peaks within the range of 2θ = 20–80° attributed to the (220), (400), (422), (511), and (533) likenesses of iron magnetite and/or maghemite. Moreover, an enhancement in the temperature of calcination from 500 to 600 °C results in a more distinct crystal structure of the product (Hamelian et al. 2023).

**Fig. 21:**
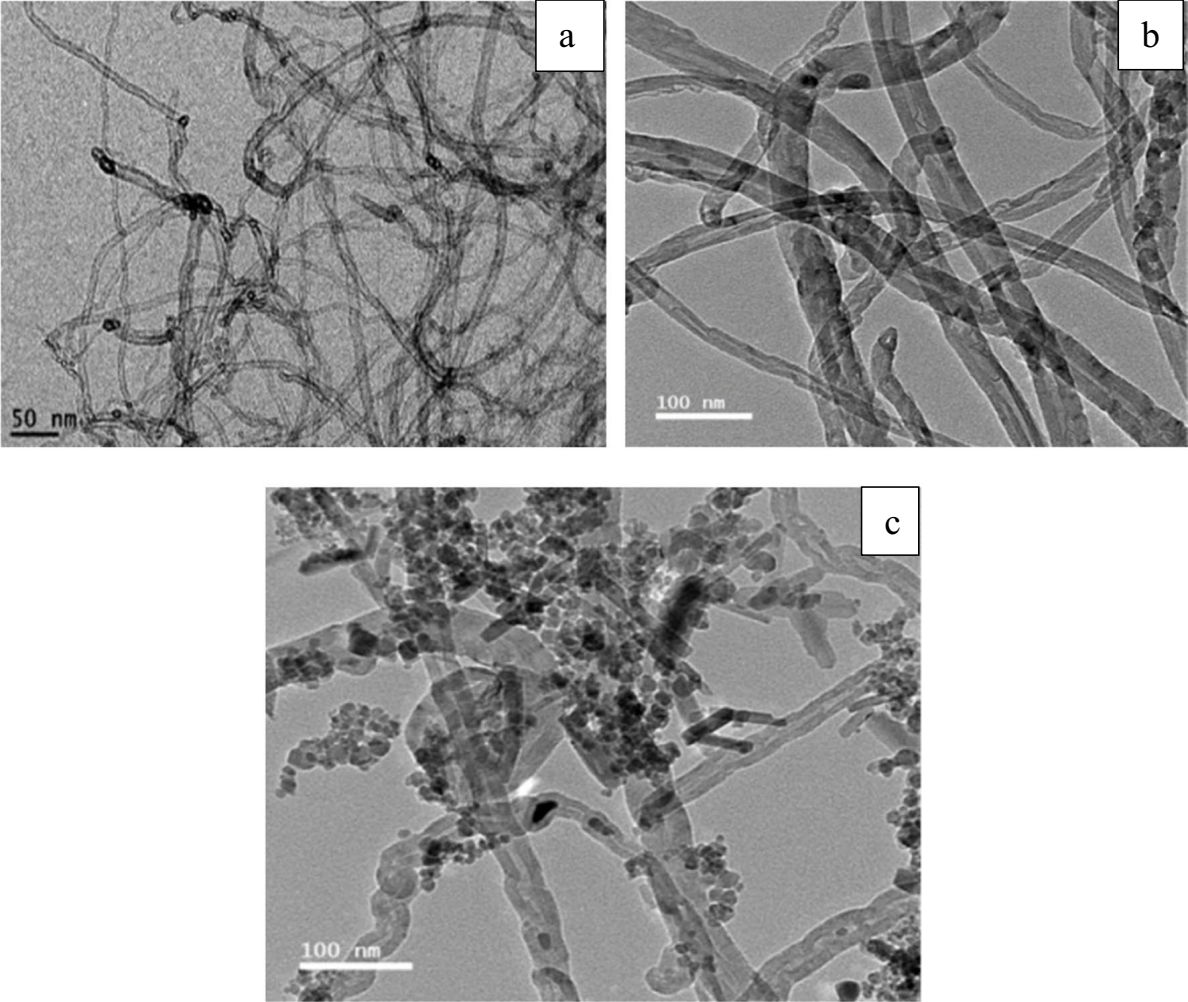
TEM images of **a** CNTs, **b** activated CNTs, and **c** magnetic CNTs

As observed in Fig. 21, the structure of pure CNTs is obviously defined. They are homogenous, with diameters varieties between 5 and 12 nm. These results also confirm that the samples analyzed are pure and free from metallic impurities and amorphous carbon deposits. The activated CNTs have not any significant difference from pure CNTs which suggests that the activation process is not affecting surface morphology (Fig. 21b). On the other hand, magnetic CNTs have visible iron particles conjugated to the tubes even after ultrasonication to scatter the CNTs/Fe_3_O_4_ complex for the characterization, the iron particles are impeded within the carbon nanotubes.

**Fig. 22:**
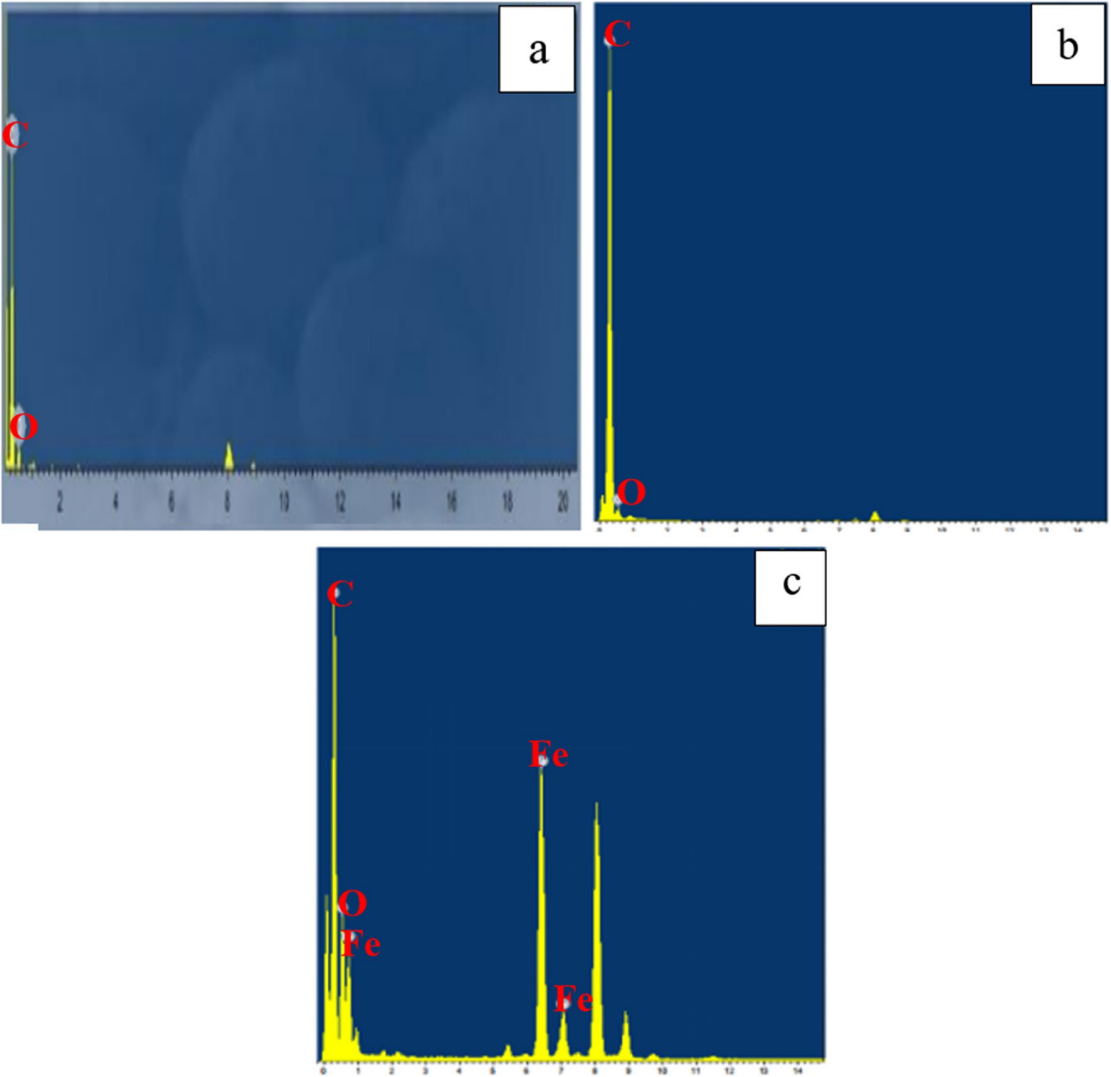
EDX analysis of **a** carbon nanotubes, **b** activated carbon nanotubes, and **c** magnetic carbon nanotubes

**Fig. 23:**
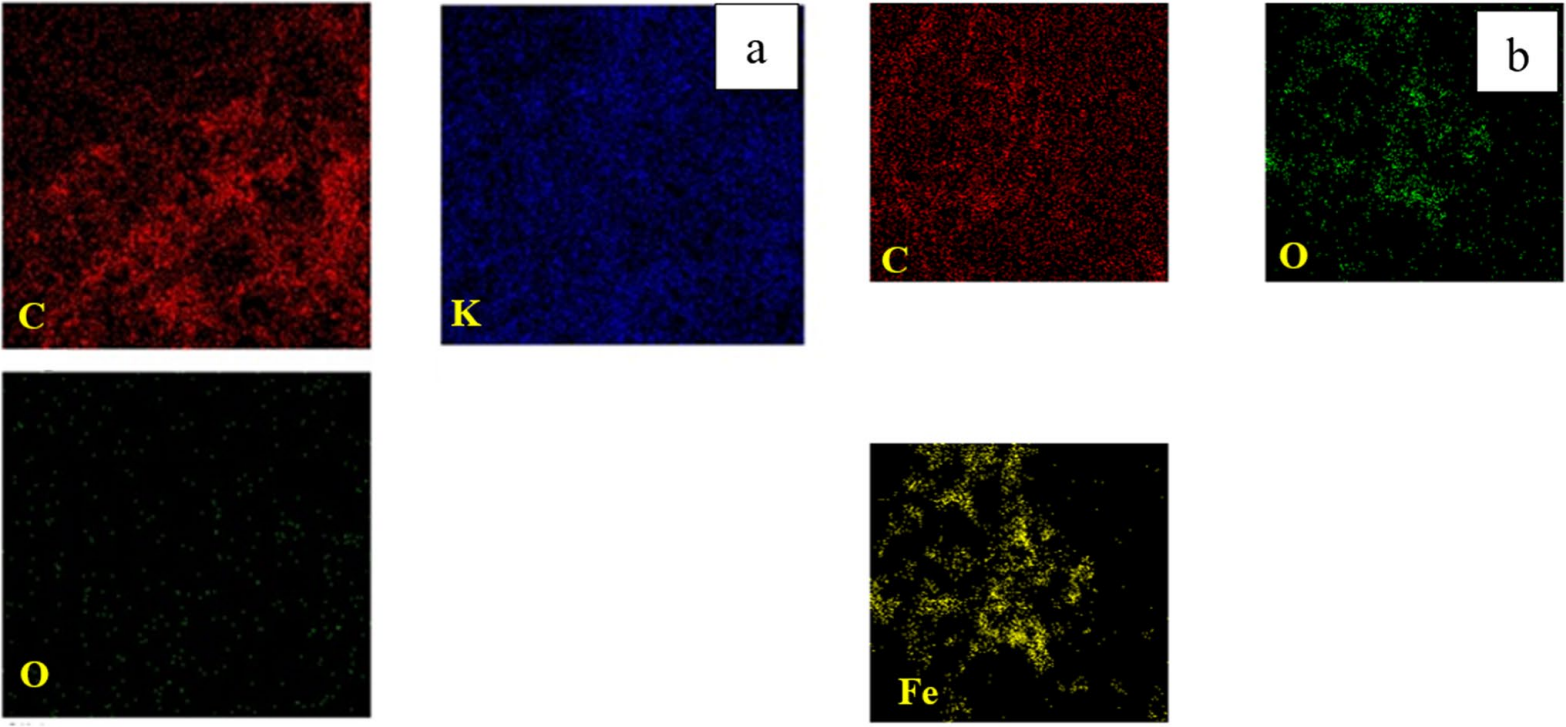
Mapping of **a** activated CNTs and **b** magnetic CNTs

The chemical analysis (EDX) performed during the TEM analysis proved that the pure CNTs contain only C and O where C= 95.17% (Figs. 22a and 23a). The same data were obtained for activated CNTs (C=98.79%) (Figs. 22b and 23b). Magnetic CNTs illustrated the attendance of Fe, O, and C in the magnetic-CNTs composition as represented in Figs. 22c and 23c. Moreover, the recorded atomic ratio of Fe and O approached 2:3, indicating the formation of γ-Fe2O3 (Ahranjani et al. 2024).

**Fig. 24:**
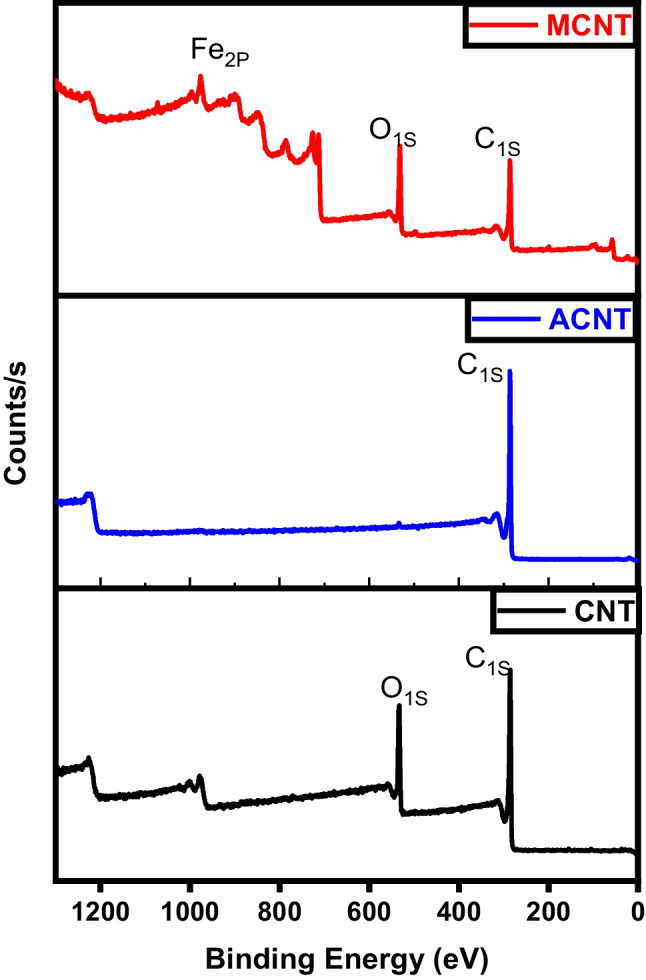
XPS analysis of CNTs, activated CNTs, and magnetic CNTs

Figure 24 displays the photoemission C_1S_ and O_1S_ peaks related to different CNTs samples. A prominent peak is noted at 284 eV, corresponding to the sp2 hybridized C=C bonds within an extensively conjugated system, indicative of graphene sheets within carbon nanotubes. Two additional significant peaks are observed at 286 and 288 eV. The peak at 286.3 eV is associated with the C–O bond, while the peak at 288.8 eV is characteristic of carbon atoms in carbonyl groups (C=O). The activated CNTs spectra reveal the absence of O_1S_ peaks. This suggests the successful elimination of the oxygenated groups during activation (Huang et al. 2019). Magnetic CNTs spectra not only reveal the existence of carbon-carbon attachment of CNTs at a binding energy of 285 eV but also indicate the construction of carbonyl moieties consistent with carboxylated groups at a binding energy of 288 eV. The nucleation spots for iron oxide were created on the surface of CNTs due to the electrostatic attraction between Fe(III) ions and the carboxylate groups on the surface of acid-treated CNTs (Ban et al. 2022).

**Fig. 25:**
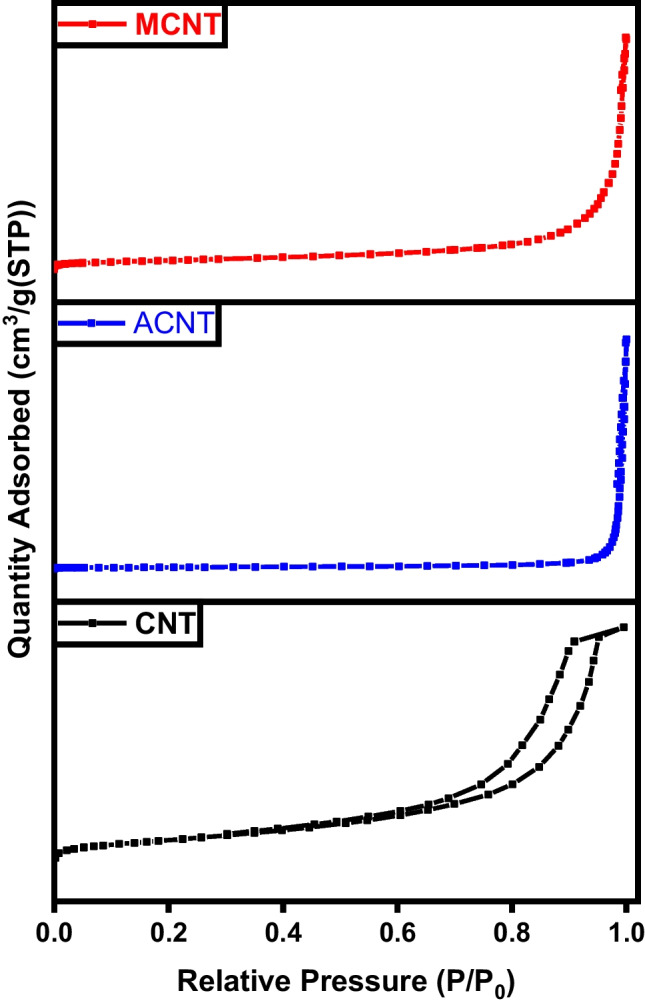
BET analysis of CNTs, activated CNTs, and magnetic CNTs

As demonstrated by the BET analysis depicted in Fig. 25, the initial carbon sample exhibited a lower surface area, which was notably enhanced through KOH activation and further augmented with sample magnetization. The adsorption/desorption hysteresis loops observed in all samples are of type IV within the relative pressure range of 0.4–0.1, indicating the successful formation of mesoporous carbonaceous materials. Moreover, the pore volume experienced a significant increase from 0.214 to 1.17 and 1.93 cm^3^ g^−1^. This increase in specific surface area and total pore volume is attributed to the reduction in H and O contents during the activation process, alongside the formation of a porous carbonic structure (Pereira et al. 2023). The surface properties of carbon nanotubes are influenced by the thickness of the tube walls. The specific surface area, total pore volume, and average pore diameter provided in Table 3 of CNT signify a boost in surface area and porosity after activation and magnetization (Elkady et al. 2020; Salama et al. 2023b).

**Table 3:** BET data of CNT samples

Sample	Specific surface area (m^2^ g^**−**1^)	Average pore diameter (nm)	Total pore volume (cm^3^ g^**−**1^)
CNT	42	14.5	1.173
ACNT	51	12	0.180
MCNT	78	28	0.550

**Fig. 26:**
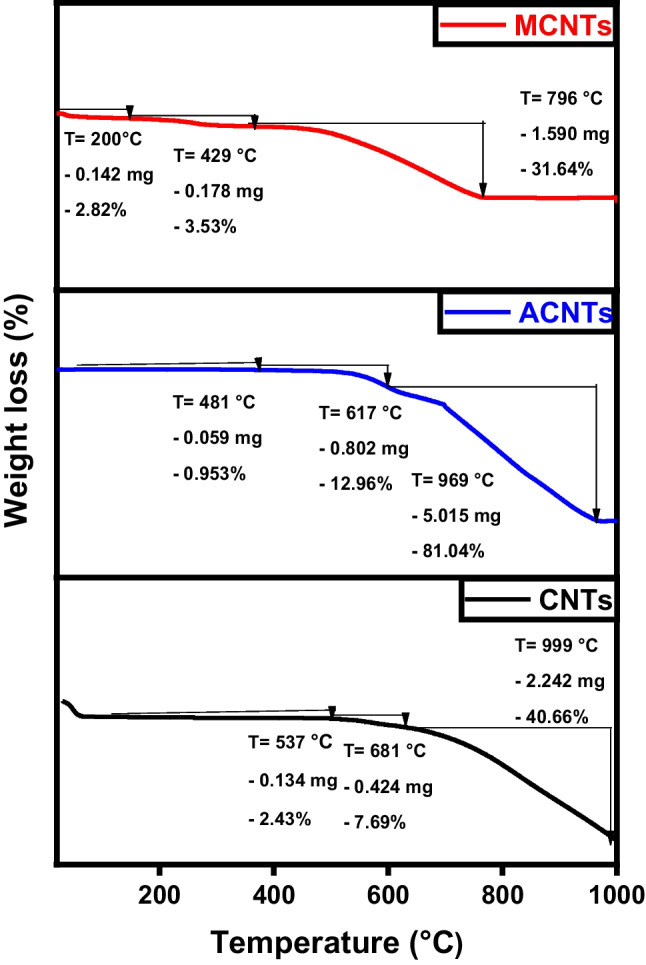
TGA analysis of CNTs, activated CNTs, and magnetic CNTs

As demonstrated in Fig. 26, both pure CNTs and activated CNTs share the same thermal behavior where most of the sample weight loss occurs between 670/680 and 999 °C. Hence, TGA analysis confirms that activation has no significant effect on chemical morphology (also proven by TEM images). For magnetic CNTs, the decline in weight noted between 50 and 200 °C was linked to the removal of water molecules from the surface of the altered CNTs, as the cause (Hu et al. 2023). The larger and more consistent reductions in weight occurring between 200 and 796 °C were associated with the pyrolytic decomposition of thiosemicarbazide (Alamdari et al. 2023).

**Fig. 27:**
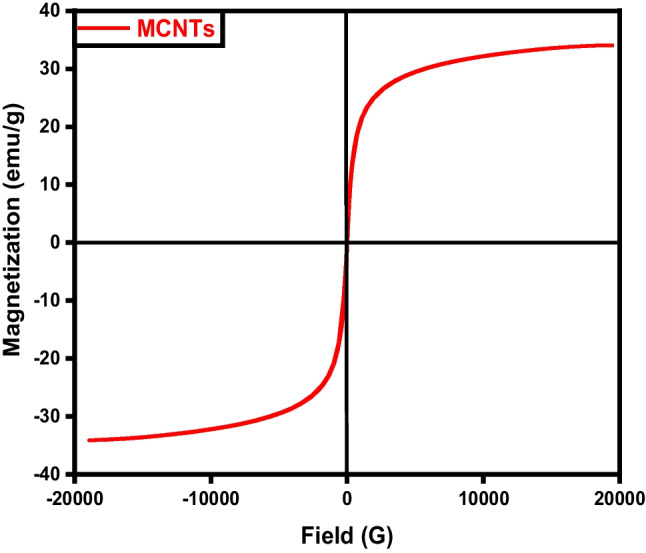
VSM analysis of magnetic CNTs

Magnetic particles can be dispersed onto the surface of carbon nanotubes and also can be incorporated within the pores. The magnetic characteristics of the MCNTs nanocomposites were evaluated via a vibrating magnetometer (Fig. 27). Analysis of the hysteresis loops indicates that the samples demonstrated a standard superparamagnetic response (Elkady et al. 2020; Elkady et al. 2017; Salama et al. 2023b). A prior investigation had described superparamagnetic behavior as the typical response of a magnetic material to an external magnetic field. The magnetic material particles exhibit a magnetization value of Ms= 27.18 emu.

### Adsorption of MB on the prepared materials

**Fig. 28:**
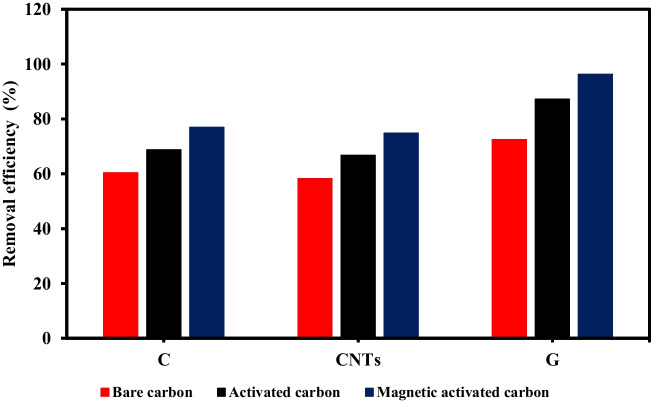
Adsorption efficiencies of MB in the case of bare, activated, and magnetic activated carbon–based materials

The adsorption efficacies of MB in the case of the pristine, activated, and magnetic-activated carbon-based materials were estimated as shown in Fig. 28 at a primary MB concentration of 10 ppm, pH 7, adsorbent dose of 1 g/L, interaction time of 180 min, and room temperature. The adsorption ratios were 76.9%, 74.8%, and 96.3% in the case of magnetically activated carbon spheres, carbon nanotubes, and graphene, respectively, whereas the removal efficiencies were 60.3%, 72.5%, and 58.2% in the case of pristine carbon spheres, graphene, and carbon nanotubes, respectively, and they were 68.7%, 87.2%, and 66.7% in the case of activated carbon spheres, graphene, and carbon nanotubes, respectively. The activated carbon–based materials exhibited higher adsorption performance compared to pure carbonaceous materials due to the improved porosity and surface area as a result of the chemical activation (Mensah et al. 2022). Moreover, the -OH surface functional group introduced by the chemical activation can improve the binding affinity for MB via hydrogen bonding and electrostatic interactions (Gotore et al. 2022; Mensah et al. 2022; Sahu et al. 2022). The highest adsorption efficiencies of MB were attained in the case of magnetic activated carbon–based materials due to the increase of surface area and porosity after magnetization (Mensah et al. 2022; Shokry et al. 2020). Magnetic activated graphene attained the highest capacity of adsorption because of its higher surface area compared to carbon spheres and CNTs materials. To confirm the effectiveness of the prepared magnetic activated graphene, its adsorption efficiency was compared with the adsorption efficiencies of other adsorbents in the literature as shown in Table 4. The comparison confirms the high performance of the prepared magnetic activated graphene.

**Table 4:** Comparison with other adsorbents in the literature

Adsorbent	Pollutant	Operating parameters	Adsorption efficiency	Reference
Modified bagasse fly ash	Malachite green and methylene blue	Dye concentration = 100 mg/L, pH = 9.6, adsorbent dose = 2 g/L, and reaction time = 51.5 min	Adsorption efficiencies = 71.5% and 67.2% for malachite green and methylene blue, respectively	
Mint-stalks derived biochar	Methylene blue	Adsorbent dose = 0.5 g/L, dye concentration = 5 mg/L, pH = 7, temperature = 30 °C, and reaction time = 90 min	Adsorption efficiency = 87.5%	
Activated waste toner powder	Methylene blue	Adsorbent dose = 0.1 g/100 mL, dye concentration = 10 mg/L, and reaction time = 3 h	Adsorption efficiency = 80%	
Fe_3_O_4_@PVA/GT	Methylene blue	pH = 9, dye concentration = 10 mg/L, temperature = 35^o^C and reaction time = 4 h	Adsorption efficiency = 93%	
Magnetic-activated graphene	Methylene blue	MB concentration =10 mg/L, pH 7, adsorbent dose = 1 g/L, and reaction time = 180 min	Adsorption efficiency = 96.3%	This study

## Conclusions

The performed analyses confirmed the chemical configuration and chemical activation of the prepared composites as well as the excellent interaction between the activated carbonaceous materials and magnetite. The activation and magnetization of bare carbon–based materials enhanced the surface area and improved the porosity. All the fabricated materials showed better resistance to thermal decomposition after magnetization. Magnetic activated carbon spheres (weight loss ratio = 21.92%) exhibited the highest thermal stability followed by magnetic activated graphene (weight loss ratio = 25.63%) and magnetic activated carbon nanotubes (weight loss ratio = 37.99%), respectively. The prepared carbonaceous materials could be easily collected after treating the aqueous solution due to their superparamagnetic features, where the magnetic saturation values were 33.77, 38.75, and 27.18 emu/g in the case of magnetic activated carbon spheres, graphene, and carbon nanotubes, respectively. The adsorption efficacies of MB were 76.9%, 96.3%, and 74.8% with respect to magnetic activated carbon spheres, graphene, and carbon nanotubes, respectively. The high adsorption performance and inexpensiveness of the prepared adsorbents provide these materials with supremacy among other materials to be applied on a larger scale.

## Data Availability

All data created or studied during this investigation are involved in this published manuscript.
